# Bioinformatics analysis to obtain critical genes regulated in subcutaneous adipose tissue after bariatric surgery

**DOI:** 10.1080/21623945.2022.2115212

**Published:** 2022-08-29

**Authors:** Shuai Chen, Yicheng Jiang, Xiaoyang Qi, Peng Song, Liming Tang, Hanyang Liu

**Affiliations:** Center of Gastrointestinal Disease, The Affiliated Changzhou NO. 2 People’s Hospital of Nanjing Medical University, Changzhou, China

**Keywords:** Obesity, bariatric surgery, adipose tissue, adipocytes, MXRA5

## Abstract

Bariatric surgery (BS) is a dependable method for managing obesity and metabolic diseases, however, the regulatory processes of lipid metabolism are still not well elucidated. Differentially expressed genes (DEGs) were analysed through three transcriptomic datasets of GSE29409, GSE59034 and GSE72158 from the GEO database regarding subcutaneous adipose tissue (SAT) after BS, and 37 DEGs were identified. The weighted gene co-expression network analysis (WGCNA), last absolute shrinkage and selection operator (LASSO) logistic regression and support vector machine-recursive feature elimination (SVM-RFE) algorithms further screened four key genes involved in the regulation of STMN2, SFRP4, APOE and MXRA5. The GSE53376 dataset was used to further confirm the differential expression of SFRP4, APOE and MXRA5 in the postoperative period. GSEA analysis reveals activation of immune-related regulatory pathways after surgery. Finally, the silencing of MXRA5 was found by experimental methods to affect the expression of PPARγ and CEBPα during the differentiation of preadipocytes, as well as to affect the formation of lipid droplets. In conclusion, SAT immunoregulation was mobilized after BS, while MXRA5 was involved in the regulation of lipid metabolism.

## Introduction

Obesity is an escalating global public health concern, with nearly one-third of the world’s population now classified as overweight or obese [1]. Obesity, one of the major components of the metabolic syndrome, is closely associated with a variety of diseases such as type 2 diabetes [2], dyslipidemia [3], cardiovascular disease [4], non-alcoholic fatty liver disease (NAFLD) [5], and certain types of cancer [6, 7]. This connection may be due to a state of low-grade chronic inflammation throughout the body, and expansion of adipose tissue (AT) may be a cause [8].

When caloric intake is excessive, adipose tissue expands in two ways: first, by differentiation of new adipocytes from progenitor cells within the tissue; second, by expansion of existing adipocytes, the latter predominating in adults who gain weight [9]. Enlarged adipocytes are usually associated with metabolic dysfunction, while small adipocytes are related to metabolic benefit [10, 11]. Although lipid storage is a natural function of adipose tissue, excessive lipid accumulation may increase the burden on adipocytes. Then, this burden may impair healthy AT function and lead to harmful complications such as metabolic stress and inflammation [12]. There is growing evidence that dysfunctional AT plays a crucial role in the pathology of insulin resistance. Meanwhile, AT is an important factor in metabolic syndrome as well as cardiovascular disease [13, 14]. In addition to AT, excessive accumulation of lipids in the liver can likewise lead to harmful metabolic complications [15].

Bariatric surgery (BS) is an important therapeutic strategy for weight loss and related metabolic syndrome relief in obese patients [16]. BS is an effective option when obese patients weigh more than 40 kg/m^2^ or weigh more than 35 kg/m^2^ with obesity-related complications [17]. One study found that the number of adipocytes remained the same after surgery, but the size of the adipocytes decreased, and the number of inflammatory immune cells within the adipose tissue was reduced [18]. In the postoperative period with massive weight loss, the subcutaneous fat inflammatory factor IL-6/TNFa decreases substantially [19, 20]. Furthermore, BS can change the status of the fat mass on the clavicle, which in turn can affect the metabolic health of the whole body [21]. But, the effects of BS on lipid metabolism still need to be further explored.

The emergence of high-throughput omics data and the development of bioinformatics have revealed a large number of potential biomarkers and patterns. Liang obtained transcriptomic datasets of ST-elevation myocardial infarction from public databases, validated key modules related to STEMI pathological status by weighted gene co-expression network analysis (WGCNA), and identified and analysed the diagnostic markers ALOX5AP and BST1 by LASSO and SVM-RFE algorithms [22]. Moreover, ARG2, MAP4K5 and TSTA3 were identified as diagnostic markers of SONFH by support vector machine-recursive feature elimination (SVM-RFE), WGCNA, last absolute shrinkage and selection operator (LASSO) logistic regression and random forest (RF) algorithms, and further validated by qRT-PCR [23]. However, most bioinformatics analyses on bariatric surgery have not been performed in combination with WGCNA and machine learning algorithms.

In this study, we analysed data from the GEO database on BS affecting transcriptome expression in subcutaneous adipose tissue. By WGCNA, LASSO logistic regression and SVM-RFE algorithms to obtain key genes regulating adipose differentiation, we further validated the regulatory role of key gene in preadipocytes.

## Materials and methods

### Data source and analysis

Four datasets related to BS affecting changes in subcutaneous adipose tissue transcriptome data were retrieved through the Gene Expression Omnibus (GEO) website (Table 1). GSE29409, GSE59034 and GSE72158 were merged by R package inSilicoMerging [24], and then the batch effects of microarray expression data were adjusted using empirical Bayesian methods [25]. Here we used the R package limma [26] for differential expression analysis to obtain differentially expressed genes (DEGs) between the different comparison groups and the control group. |Log (fold change, FC)|>1 and *P* < 0.05 were considered as DEGs. The GSE53376 dataset was used for validation of the subsequent analysis.

**Table 1.** Data resource.

ID	Status	Organism	Tissue
GSE29409	pre-BS (n = 5), post-BS (n = 5)	Homo sapiens	Subcutaneus adipose tissue
GSE59034	pre-BS (n = 16), post-BS (n = 16)	Homo sapiens	Subcutaneus adipose tissue
GSE72158	pre-BS (n = 42), post-BS (n = 42)	Homo sapiens	Subcutaneus adipose tissue
GSE53376	pre-BS (n = 16), post-BS (n = 16)	Homo sapiens	Subcutaneus adipose tissue

### Function enrichment analysis

For gene set functional enrichment analysis we used Gene Ontology (GO) annotations of genes from the R package org.Hs.eg.db (version 3.1.0) and obtained kegg gene annotations using the Kyoto Encyclopaedia of Genes and Genomes (KEGG) rest API (https://www.kegg.jp/kegg/rest/keggapi.html), respectively. Using this as a background, the genes were mapped to the background set and enrichment analysis was performed using the R package clusterProfiler (version 3.14.3) to obtain the results of gene set enrichment.

### Screening and confirmation of biomarkers

A co-expression network was formed through the WGCNA R package [27] in order to find the most relevant modules to the phenotype. First, missing values and outliers are processed. The network was constructed by a one-step method. Correlate module data with phenotypic data to obtain the genes in the module with the strongest correlation to surgery for the next step of analysis. Further analysis of DEGs by the LASSO logistic regression and SVM-RFE was performed to obtain key genes for surgical impact on SAT. ‘glmnet’ and ‘e1071’ R packages are applied respectively [28,29]. The LASSO algorithm is performed by 10-fold cross-validation to adjust the optimal value of the penalty parameter λ. SVM‐RFE algorithm searching for lambda with the smallest classification error to determine the variable.

### Gene set enrichment analysis (GSEA)

We obtained the GSEA software (version 3.0) from the GSEA website (http://software.broadinstitute.org/gsea/index.jsp), divided the samples into two groups based on preoperative and postoperative status. The c2.cp.kegg.v7.4.symbols.gmt subset was downloaded from the Molecular Signatures Database (http://www.gsea-msigdb.org/gsea/downloads.jsp) to evaluate related pathways and molecular mechanisms, based on gene expression profiles and phenotypic groupings, with a minimum gene set of 5 and a maximum gene set of 5000, and one thousand resamplings, with P values of < 0.05 and a false discovery rate (FDR) of < 0.25 were considered statistically significant.

### Cell type enrichment analysis

xCell was used to assess the distribution of 64 immune and stromal cell species in tissues, based on gene expression profile [30]. For each cell type, xCell abundance scores were calculated in four main steps: (i) ssGSEA was performed individually for 489 gene sets using the R package GSVA; (ii) ES was averaged across all gene sets belonging to a cell type; (iii) platform-specific ES was converted to abundance scores; (iv) similar to that used for flow cytometry data analysis using the spillover method to correct for correlations between closely related cell types. Therefore, raw enrichment scores and immune scores were emerged.

### Preadipocytes extraction and culture

Adipose tissue was taken from the visceral tissue of obese patients undergoing BS and repeatedly rinsed using phosphate-buffered saline (PBS). Blood vessels and connective tissue were removed using scissors, and the fat was processed into a minced meat form. The tissue was then digested in 0.1% type I collagenase (Gibco) for 30 min. After 30 min, the tissue was filtered through 200 mesh screen, then centrifuged to remove the liquid and cultured in DMEM/F12 medium (Gibco) with 10% foetal bovine serum (FBS, Gibco), 100 U/ml penicillin and 100 μg/ml streptomycin complete medium, incubated at 37°C in a 5% CO_2_ incubator, and the medium was changed once every 2 days, while cell density was observed. After the cell density reached about 60–80%, 1:2 passages were made and the culture was continued. The Affiliated Changzhou NO. 2 People’s Hospital of Nanjing Medical University ethics committee approved the study (Ethics Approval Number YLA001).

### Lipogenic differentiation of preadipocytes

Cells were inoculated into culture dishes and when the cells reached 100% confluence, complete medium containing 1 μmol Dexamethasone (Aladdin, China), 0.5 mmol 3-isobutyl-1methylxanthine (IBMX, Aladdin, China) and 10 μg/ml insulin (Aladdin, China) was added to induce differentiation for 48 h. After 48 h, complete medium containing 10ug/ml insulin was changed to continue to induce differentiation for 2 days, and complete medium culture until cells are required for the next experimental step.

### Oil red O staining

Oil Red O powder (Sigma) was dissolved in isopropyl alcohol and diluted 3:2 using distilled water to form a working solution, which was filtered through filter paper and used. Cells were fixed with 4% polymethanol for 30 minutes, were washed 3 times with PBS, then were stained with Oil Red O working solution in a dark room for 30 minutes, and finally were observed and photographed under a microscope.

### RNA isolation and quantitative real-time polymerase chain reaction (qRT-PCR)

Total RNA was extracted from the cells using Trizol, as described in the instructions, and reverse transcribed using Vazyme HiScript II Q RTSuper mix (Vazyme, China). The RT-PCR reaction was performed as follows: 37°C for 5 min, 85°C for 5 sec and finally reduced to 4°C. qPCR was performed using AceQ qPCR SYBR Green Master Mix (Vazyme, China). The reaction was conducted as follows: 95°C for 5 min, followed by 40 cycles at 95°C for 10 sec and 60°C for 30 sec. The mRNA expression level for each target gene was normalized to the level of GAPDH. The primers used were listed in Table 2. The fold changes in expression of each gene were calculated by 2^−(ΔΔCt)^ method.

**Table 2.** Primer sequences.

Gene	Sequences (5’-3’)
MXRA5 F	CCTTGTGCCTGCTACGTCC
MXRA5 R	TTGGTCAGTCCTGCAAATGAG
PPARγ F	GCCCTTCACTACTGTTGACTTCTCC
PPARγ R	CAGGCTCCACTTTGATTGCACTTTG
CEBPα F	TCGGTGGACAAGAACAGCAACG
CEBPα R	GGCGGTCATTGTCACTGGTCAG
GAPDH F	CATGTTCCAATATGATTCCAC
GAPDH R	CCTGGAAGATGGTGATG

### Western blotting

Total protein extraction was performed with RIPA buffer (Beyotime, China) containing 1 mM PMSF (Beyotime, China). Protein extracts were subjected to SDS-PAGE with 10%, and then transferred onto PVDF membranes (Beyotime, China) . Blocking of the membranes was performed for two hours with blocking buffer (Beyotime, China). Membranes were incubated in antibody CEBPα (Abmart, China), PPARγ (Abmart, China) and GAPDH (Proteintech, China) respectively overnight at 4°C. Afterwards, the membranes were incubated with a horseradish peroxidase (HRP)–coupled secondary antibody (Proteintech, China). Detection of immunoreactive bands was carried out by using the ECL Western Blot Detection Reagents from Thermo Scientific (SuperSignal West Pico Chemiluminescent Substrate).

### Cell transfection

Small interfering RNAs (siRNA) of MXRA5 were purchased from RIBIO. Operation was calculated according to manufacturer’s protocol. Cells were first inoculated in 6-well plates, and when cell confluency reached 60%-80%, siRNA (concentration 50 nmol) was transfected according to the instructions. After 24 hours, the cells were subjected to lipogenic induction of differentiation for 48 hours. Then, a second siRNA transfection was performed, and 12 hours later, differentiation was induced by changing the medium containing insulin until differentiation into mature adipocytes.

### Statistical analysis

SPSS 23.0 (SPSS statistics, USA) was used for general statistical analysis. GraphPad Prism 8.0 software (GraphPad Software, USA) and R Studio were used to generate plots. Statistical differences between groups were tested using Student’s t-test. Spearman correlation analysis was performed to calculate correlation coefficients. Data are presented as the mean ± SD values. ‘P < 0.05’ commonly indicates a statistically significant difference. The cell experiment was repeated three times.

## Results

1.DEGs after bariatric surgery

**Figure 1.**
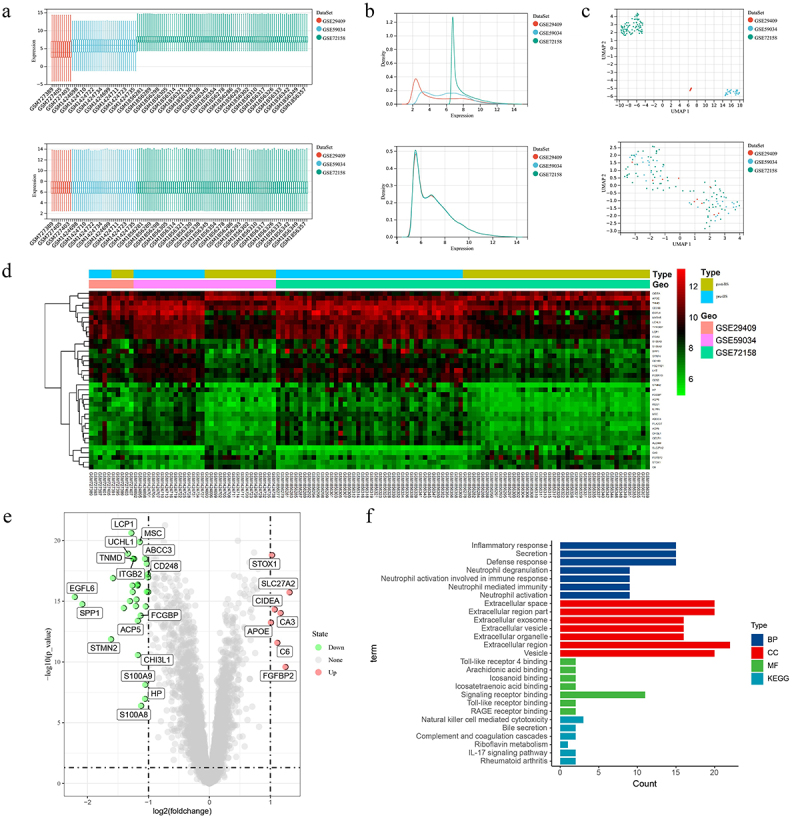
Data merging, analysis and feature enrichment. (a, b, c) After removing the batch effect the data distribution tends to be consistent across datasets with similar means and variances, and the samples are clustered and intertwined with each other between datasets. (d) Heat map of 37 DEGs was obtained from the analysis of the merged data. (e) Green circles represent 30 down-regulated DEGs and red circles represent 7 up-regulated DEGs in the volcano plot. (f) GO and KEGG analysis of all DEGs. DEGs, differentially expressed genes; GO, Gene Ontology; KEGG, Kyoto Encyclopaedia of Genes and Genomes.

Combining the three datasets GSE29409, GSE59034 and GSE72158, after removing the batch effect, the distribution of data among the datasets tends to be consistent, with the median on a line (Figure 1(a)), the mean and variance similar (Figure 1(b)), and the samples clustered together among the datasets (Figure 1(c)), suggesting a better removal of the batch effect. Setting |logFC|>1 and *P* < 0.05 as screening conditions, we observed 7 upregulated DEGs and 30 downregulated DEGs in SAT after BS (Figure 1(d-e) and Table 3).

**Table 3.** Differentially expressed genes after bariatric surgery.

Differentially expressed genes	Genes name
Up-regulated	APOE STOX1 CIDEA C6 CA3 FGFBP2 SLC27A2
Down-regulated	EGFL6 SPP1 STMN2 PLA2G7 LYZ UCHL1 CD52 LCP1 MXRA5 AQP9 TNMD ITGB2 SFRP4 IL1RN RGS1 ALCAM ACP5 CHI3L1 MSC FCGBP S100A8 ABCC3 HP S100A9 HSD11B1 CECR1 FCER1G CD248 TYROBP CD163

### DEGs enrichment analysis

Understanding the role of all DEGs in biology through GO and KEGG analysis (Figure 1(f)). For biological process (BP), mainly related to inflammatory response, secretion, defence response, neutrophil degranulation and neutrophil activation involved in immune response. For cellular component (CC), it mainly contains extracellular space, extracellular region part, extracellular exosome, extracellular vesicle and extracellular organelle. For molecular function (MF), Toll-like receptor 4 binding, arachidonic acid binding, icosanoid binding, icosatetraenoic acid binding and signalling receptor binding were significantly enriched. The enriched KEGG mainly includes 6 pathways (natural killer cell mediated cytotoxicity, bile secretion, complement and coagulation cascades, riboflavin metabolism, IL-17 signalling pathway and rheumatoid arthritis).

### Identification of post-BS related genes using WGCNA

**Figure 2.**
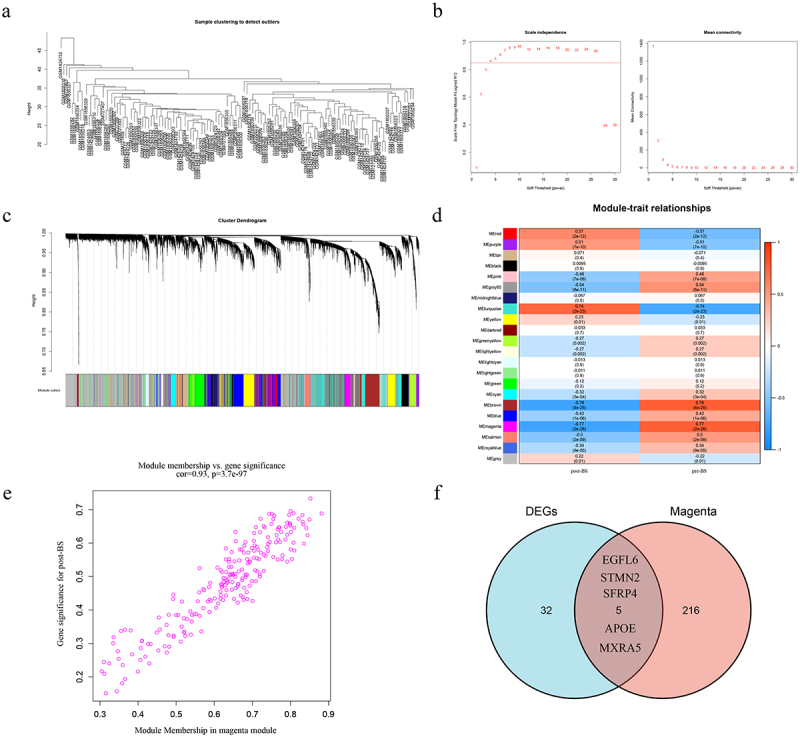
WGCNA acquires DEGs associated with surgery. (a) Determine the number of genes and samples, and then construct a sample clustering tree. (b) Analysis of the scale-free fit index and the mean connectivity for various soft-thresholding powers. (c) A one-step network construction generated 22 modules. (d) Module and BS correlation heat map. (e) The gene significance for BS in the magenta module. (f) Venn diagram showing genes shared by DEGs and magenta modules. WGCNA, weighted gene co-expression network analysis; DEGs, differentially expressed genes; BS, bariatric surgery.

Screening for core genes using WGCNA. First filtered out the missing values and kept 126 samples and 10,032 genes to form a sample clustering tree (Figure 2(a)). The R function pickSoftThreshold is used to calculate the soft threshold power β. When the soft threshold power β is set to 4, the scale independence reaches 0.9 and has a relatively high average connectivity (Figure 2(b)). Then 22 modules were generated by one-step network construction (Figure 2(c)). We found that the MEmagenta module had the highest preoperative and postoperative correlation, with 221 genes contained within the MEmagenta module (Figure 2(d)). The importance of the gene set in MEmagenta after surgery was also analysed (Figure 2(e)). Analysis of the genes in the MEmagenta module revealed that five genes (EGFL6, STMN2, SFRP4, APOE and MXRA5) were DEGs (Figure 2(f)). The results showed that these five genes were significantly associated with postoperative SAT changes.

### Optimal biomarker identification

**Figure 3.**
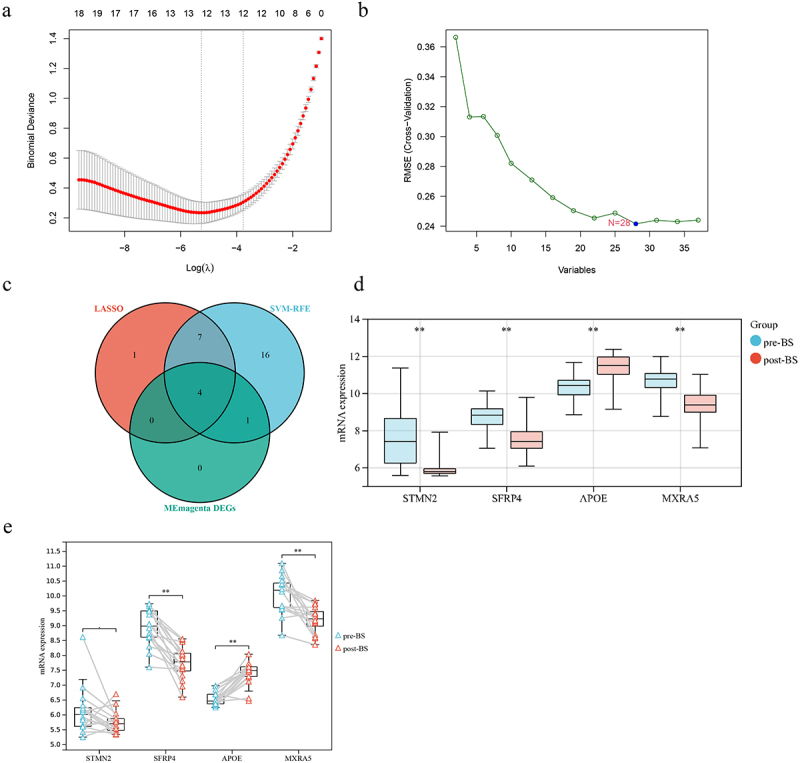
Screening for post-BS-related markers by a comprehensive strategy. (a) The lasso regression analysis identified 12 genes with minimum lambda values and non-zero parameters. (b) SVM-RFE used the minimum RMSE to identify 28 genes. (c) Intersection of LASSO, SVM-RFE, and magento modules for DEGs. (d) Differential expression of STMN2, SFRP4, APOE and MXRA5 in the combined dataset (e) Validation of STMN2, SFRP4, APOE and MXRA5 expression in GSE53376. Student’s t-test was used to compare between the two groups. *P < 0.05; **P < 0.01. BS, bariatric surgery; SVM-RFE, support vector machine-recursive feature elimination; RMSE, root mean square error; DEGs, differentially expressed genes.

DEGs were further screened using both LASSO logistic regression and SVM-RFE algorithms to obtain 12 and 28 significant pivotal genes, respectively (Figure 3(a-b)). Four important DEGs, STMN2, SFRP4, APOE and MXRA5, were obtained under three different analyses of LASSO, SVM-RFE and WGCNA (Figure 3(c)). Differences in the expression of STMN2, SFRP4, APOE and MXRA5 were examined in the combined dataset (Figure 3(d)). Moreover, in the external dataset GSE53376 to validate the differential expression of STMN2, SFRP4, APOE and MXRA5, similar results were found for SFRP4 and MXRA5 which were downregulated postoperatively, while APOE was upregulated (Figure 3(e)). Results demonstrate that SFRP4, APOE and MXRA5 are pivotal genes involved in the regulation of SAT after BS.

### Immunomodulation and immune cell infiltration after BS

**Figure 4.**
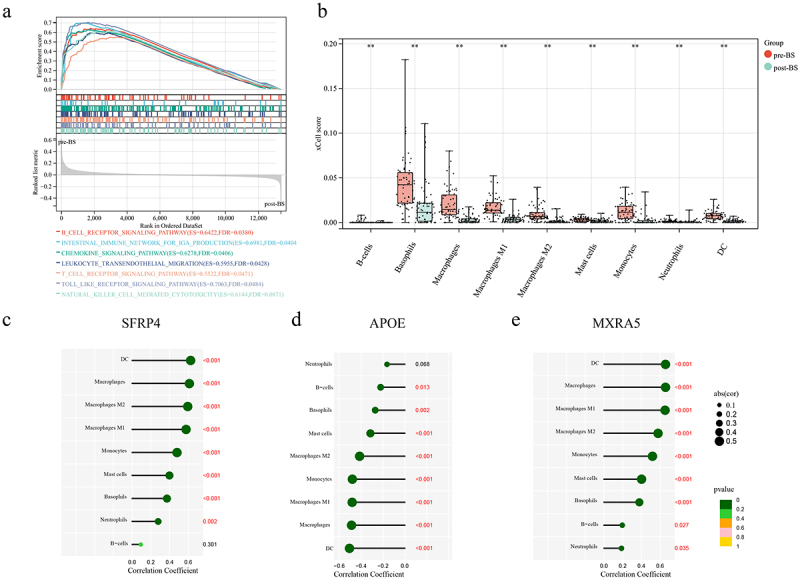
Assessing the role of immunomodulation in bariatric surgery. (a) GSEA analysis of the effect on immune pathways after bariatric surgery. (b) xCell analysis results to observe the altered level of immune cell infiltration after surgery. (c, d, e) Correlation of SFRP4, APOE and MXRA5 expression levels and the degree of immune cell infiltration. Student’s t-test was used to compare between the two groups. *P < 0.05; **P < 0.01. Spearman correlation analysis was performed to calculate correlation coefficients. GSEA, Gene set enrichment analysis.

The results of GSEA analysis showed that B cell receptor signalling pathway, intestinal immune network for IGA production, chemokine signalling pathway, T cell receptor signalling pathway, toll like receptor signalling pathway and natural killer cell mediated cytotoxicity were significantly enriched (Figure 4(a)). Further analysis of changes in immune cell infiltration levels after surgery was performed through the xCell online analysis website. The results showed that there were significant changes in B-cells, Basophils, Macrophages, Macrophages M1, Macrophages M2, Mast cells, Monocytes, Neutrophils and dendritic cells (DCs) in the postoperative period (Figure 4(b)). Correlation analysis SFRP4 expression was significantly and positively correlated with the degree of DCs, Macrophages, Macrophages M2, Macrophages M1, Basophils, Monocytes, Mast cells, and Neutrophils infiltration (Figure 4(c)). However, the respective B-cell correlation was not statistically significant. The expression of APOE was significantly and negatively correlated with the degree of DCs, Macrophages, Macrophages M1, Monocytes, Macrophages M2, Mast cells, Basophils and B-cells infiltration (Figure 4(d)). However, the respective neutrophil correlation was not statistically significant. In addition, the expression of MXRA5 was significantly and positively correlated with the degree of DCs, Macrophages, Macrophages M1, Macrophages M2, Monocytes, Mast cells, Basophils, B-cells and Neutrophils infiltration (Figure 4(e)).

### Silencing MXRA5 expression inhibits differentiation of preadipocytes

**Figure 5.**
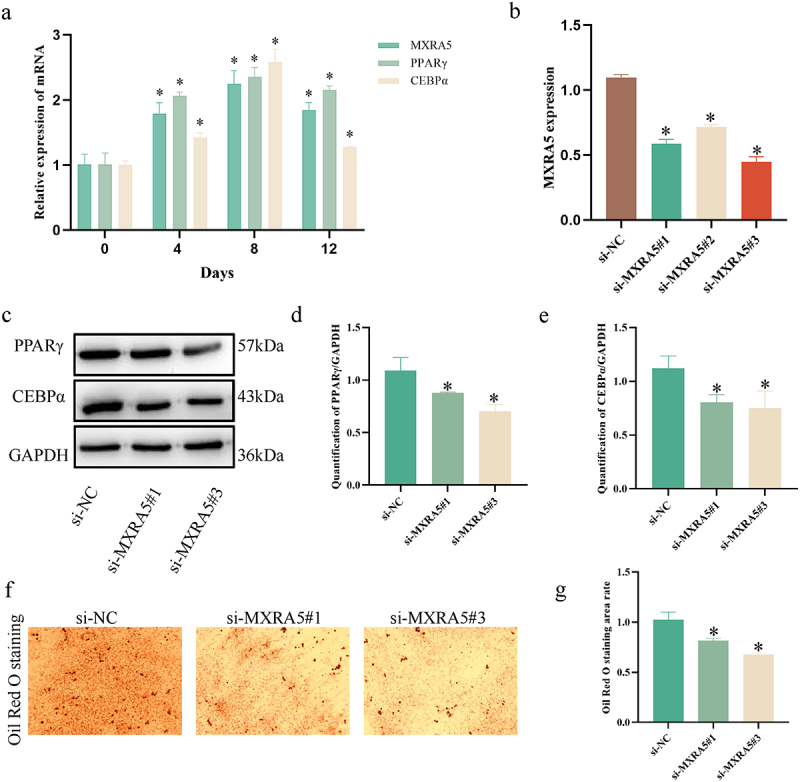
Experimental validation of MXRA5 function in adipocyte maturation. (a) Expression levels of MXRA5, PPARγ and CEBPα in preadipocytes during adipogenic differentiation. *P < 0.05, compared to 0 days. (b) Validation of MXRA5 transfection efficiency. *P < 0.05, compared to si-NC. (c, d, e) Silencing MXRA5 reduced the protein expression levels of PPARγ and CEBPα. (f, g) Silencing MXRA5 reduces lipid droplet formation. Student’s t-test was used to compare between the two groups. *P < 0.05.

Based on the collation of literature, no exploration of MXRA5 in adipocytes was found, so the function of MXRA5 in adipocytes was experimentally verified in this study. During the induction of lipogenic differentiation in preadipocytes, the expression levels of MXRA5, PPARγ and CEBPα were significantly increased on days 0, 4, 8 and 12 (Figure 5(a)). The results suggest that MXRA5 is involved in the regulation of lipogenic differentiation of preadipocytes. The silencing efficiency of si-RNA was verified by qPCR (Figure 5(b)), and si-MXRA5#1 and si-MXRA5#3 silencing effects were relatively good, so both were selected for follow-up experiments. The effect of silencing MXRA5 on adipocytes was observed at day 8 of induction of differentiation. We found that silencing MXRA5 expression affected the alteration of PPARγ and CEBPα protein levels (Figure 5(c-e)) and reduced the formation of lipid droplets (Figure 5(d, g)). This suggests that MXRA5 is involved in the regulation of lipid maturation during.

## Discussion

Currently, BS is the most effective treatment for morbid obesity and its complications [31]. The main effect of bariatric surgery on is considered to be the rebalancing and metabolic recovery [32, 33]. In this study, by mining the gene expression data of subcutaneous adipose tissue microarrays of obese patients before and after BS and performing related bioinformatics analysis, we can further understand the potential mechanism of weight loss in improving metabolism, in order to discover some key factors in the process of obesity development and provide some reference for clinical treatment.

We identified 37 differentially expressed genes by merging and analysing the datasets. Enrichment analysis of differential genes revealed that BS affected inflammatory phenotypes in adipose and biological processes regulated by neutrophils. When xCell analysed the degree of immune cell infiltration, it also revealed a significant reduction in immune scores of neutrophils after BS, which showed similar results to Dulfary’s study [34]. In the KEGG pathway enrichment, three immune system-related pathways, Natural killer cell mediated cytotoxicity, Complement and coagulation cascades and IL-17 signalling pathway, were significantly enriched. The immune system changes in adipose tissue after BS were further confirmed using GSEA analysis, and several immune system-related pathways were found to be activated after surgery (Figure 4(a)). B cell receptor signalling pathway, chemokine signalling pathway, T cell receptor signalling pathway, toll like receptor signalling pathway and natural killer cell mediated cytotoxicity, these pathways have been shown to play an important role in the regulation of adipose inflammation [32–35].

In obese individuals, accumulation of adipose tissue macrophages (ATM) is associated with adipocyte and metabolic dysfunction [36]. In the normal weight state, the balance of the macrophage population tends to shift towards the anti-inflammatory M2-like subpopulation, but in the obese state, the balance shifts towards the M1-like subpopulation, creating an inflammatory environment in the adipose tissue [37]. Several studies have found significant reductions in ATM one year after weight loss surgery [20, 38]. Meanwhile, a more convergent macrophage phenotype towards M2-like macrophage transformation was observed after BS [18]. The results of our analysis are generally consistent with previous studies, reflecting the importance of immune cell regulation in adipose tissue, where unarticulated mechanisms need to be further explored.

Secretory frizzled-related protein (SFRP) 4 is an extracellular antagonist of Wnt signalling and regulates adipogenesis [39]. Overexpression of recombinant active SFRP4 protein promotes brown adipocyte differentiation and induces CEBPα, UCP-1, PRDM16, PGC1α and GLUT4 expression in a dose-dependent manner [40]. However, SFRP4 is differentially expressed in the subcutaneous and visceral adipogenic differentiation of C57BL/6 mice and may play different roles [41]. SFRP4 is also involved in the regulation of hepatic lipid accumulation and insulin resistance [42]. Meanwhile, SFRP4 expression levels were significantly elevated in obese and type 2 diabetic patients, and increased β-cell dysfunction and suppressed insulin secretion [43]. Altered expression of SFRP4 in the postoperative period may be one of the reasons why BS affects weight loss and insulin sensitivity.

Apolipoprotein E (APOE) is known to be essential in the catabolism of lipoproteins. Although APOE KO mice are protected against diet-induced obesity, it spontaneously develops severe hypercholesterolemia and atherosclerotic lesions [44]. Significant amounts of APOE are expressed and secreted in adipocytes, where expression is induced during differentiation, while APOE is undetectable in preadipocytes [45]. Adipocytes isolated from APOE-/- mice exhibit smaller adipocytes showing reduced expression of lipogenic genes and lower triglyceride and fatty acid content [46]. However, in our analysis, APOE was consistently highly expressed in postoperative subcutaneous adipose tissue, which contradicts the weight loss and reduced body fat after BS, which may be associated with APOE being polymorphic. Farup et al. found that weight loss after bariatric surgery was not associated with APOE allele variability [47], whereas a subsequent study found a positive correlation between E4 and lipocalin in a cohort of patients after bariatric surgery, which may have a role in influencing postoperative weight, and suggested that the association between APOE genotype and endocrine function may indicate that weight change is a metabolic effect rather than a direct effect of APOE direct effect of genotype [48]. The role of APOE polymorphisms in obese patients or post-bariatric surgery patients may require a large number of clinical samples or animal studies to explore its role and value, and deserves further attention.

Secreted glycoprotein Matrix-remodelling associated protein 5 (MXRA5) is an important member of the MXRA protein family that plays a role in cell adhesion and extracellular matrix remodelling (ECM) [49]. MXRA5 is mainly expressed in the extracellular matrix and cytoplasm, and can be detected in body fluids such as joint fluid and urine [50, 51]. In our study, MXRA5 was found to be diminished in subcutaneous adipose tissue by BS regulation, while silencing of MXRA5 was found to affect the expression of adipose maturation markers as well as to affect lipid droplet formation in cellular level studies. Moreover, when human-derived white fat is cultured in vitro, MXRA5 is secreted in a time-dependent manner [52]. Therefore, our study may contribute to the understanding of the molecular mechanisms affecting lipid metabolism after BS.

Our study also has some shortcomings and limitations. First, the clinical information was imperfect, lacking specific data on age, sex and weight. A second limitation of this study is that it relied on public databases and published articles for data sources. This may have influenced the results in some way due to the quality of the data. Third, the choice of statistical methods and the accuracy of the databases used for data analysis could have an impact on the interpretation of the study’s findings. In spite of this, we obtained similar results from analysing multiple databases and performing experimental validations, supporting the findings we have made.

## Conclusion

In summary, we identified 37 genes the expression of which was found to be altered in subcutaneous adipose tissue after BS. Moreover, three key biomarkers, SFRP4, APOE and MXRA5, were identified by WGCNA, LASSO and SVM-RFE algorithms. Meanwhile, we found that postoperative immunomodulatory pathways were activated, M1 macrophages and neutrophils were significantly downregulated, and SFRP4, APOE and MXRA5 were significantly correlated with the degree of immune cell infiltration. Finally, cellular experiments demonstrated the involvement of MXRA5 in the regulation of precursor adipocyte differentiation.

## Data Availability

Detailed data on this study can be found in GEO database (http://www.ncbi.nlm.nih.gov/geo), reference numbers [GSE29409, GSE59034, GSE72158, and GSE53376].
